# Potent T cell agonism mediated by a very rapid TCR/pMHC interaction

**DOI:** 10.1002/eji.200636743

**Published:** 2007-03

**Authors:** Jonathan M Boulter, Nicole Schmitz, Andrew K Sewell, Andrew J Godkin, Martin F Bachmann, Awen M Gallimore

**Affiliations:** 1 Department of Medical Biochemistry and Immunology, Cardiff University School of MedicineCardiff, UK; 2 Cytos Biotechnology AGZurich, Switzerland

**Keywords:** Biophysics, Protein-protein interactions, TCR

## Abstract

The interaction between T cell receptors (TCR) and peptide-major histocompatibility complex (pMHC) antigens can lead to varying degrees of agonism (T cell activation), or antagonism. The P14 TCR recognises the lymphocytic choriomeningitis virus (LCMV)-derived peptide, gp33 residues 33–41 (KAVYNFATC), presented in the context of H-2D^b^. The cellular responses to various related H-2D^b^ peptide ligands are very well characterised, and P14 TCR-transgenic mice have been used extensively in models of virus infection, autoimmunity and tumour rejection. Here, we analyse the binding of the P14 soluble TCR to a broad panel of related H-2D^b^-peptide complexes by surface plasmon resonance, and compare this with their diverse cellular responses. P14 TCR binds H-2D^b^-gp33 with a K_D_ of 3 µM (±0.5 µM), typical of an immunodominant antiviral TCR, but with unusually fast kinetics (*k*_off_=1 s^−1^), corresponding to a half-life of 0.7 s at 25°C, outside the range previously observed for murine agonist TCR/pMHC interactions. The most striking feature of these data is that a very short half-life does not preclude the ability of a TCR/pMHC interaction to induce antiviral immunity, autoimmune disease and tumour rejection.

## Introduction

The interaction between a T cell receptor (TCR) and an MHC-peptide complex (pMHC) underpins the biological nature of any given T cell response [1]. This interaction impinges upon the development of T cells in the thymus, thereby shaping the repertoire of T cells entering the peripheral pool. Relatively high affinity/avidity interactions (affinity being a measurable biophysical parameter, avidity being a more loosely used term to describe multivalent interactions between cells) with self pMHC in the thymus result in death of immature double-positive T cells, a process known as negative selection [2], whereas low affinity/avidity interactions result in survival and differentiation of immature thymocytes into single-positive mature T cells which enter the periphery, a process known as positive selection. Although high-affinity interactions between TCR and pMHC complexes cause death of thymocytes, similar interactions result in activation of mature T cells in the periphery. On the other hand, positively selecting ligands do not activate mature T cells, although low-affinity interactions between mature T cells and self pMHC complexes are essential for the maintenance of the peripheral T cell pool [3].

In the periphery, the type of response elicited in a given T cell by different pMHC complexes is varied. Within the CD8^+^ T cell population, some peptides, known as strong agonists, are potent inducers of T cell activation. Such peptides are efficient at inducing TCR down-regulation, T cell proliferation and acquisition of effector function (including cytotoxic activity) in response to a range of peptide concentrations. Weak agonist peptides induce TCR down-regulation, T cell proliferation and effector function inefficiently and only in response to high peptide concentrations, whilst partial agonists may stimulate some TCR down-regulation and proliferation but fail to induce effector function [4]. Some weak or partial agonists also demonstrate antagonist activity characterised by *in vitro* inhibition of lysis of target cells pulsed with agonist peptides.

Many studies have focused on identifying factors that determine the result of an encounter between a given TCR and various pMHC complexes. It is accepted that TCR bind MHC peptide complexes with very low affinity (1–50 µM) and rapid on-off kinetics at 25°C (seconds). Several groups have reported that strong agonists display higher affinities and slower dissociation rates than weaker agonists and antagonists. These data support a kinetic proofreading model where the principle binding parameter controlling T cell activation is the TCR off-rate or mean dwell-time. Stronger TCR/pMHC interactions generally give better T cell responses [5]. There are exceptions, however, and antagonist peptides from some studies have been shown to exhibit slower TCR dissociation rates than agonist peptides from other studies [6]. It is also possible that multiple TCR could interact sequentially with pMHC molecules (serial triggering model), thereby increasing T cell sensitivity [7]. In this model, the TCR has an optimal mean dwell-time beyond which T cell sensitivity is not increased because optimal signalling occurs by multiple TCR interacting in series with limiting specific pMHC. More recent data implies that discrepancies such as those described above can be accounted for by changes in conformational heat capacity (Δ*Cp*_conf_), a parameter that reflects the change in the conformation of the TCR and that, as well as half-life, contributes to the stimulatory potential of the TCR/pMHC interaction [8]. Δ*Cp*_conf_ cannot be measured directly unless structures of bound and unbound TCR are available, but Δ*Cp* (the total heat capacity change for the binding reaction) can be measured from a plot of Δ*G*^°^ (the free energy change for the binding reaction) against temperature, and this provides a limit for Δ*Cp*_conf_.

The P14 TCR is specific for the H–2D^b^-gp33 peptide (KAVYNFATC) derived from lymphocytic choriomeningitis virus (LCMV). P14 TCR-transgenic mice have been extensively used for analysing T cell responses to the index peptide, gp33, and a range of altered peptide ligands (APL), including antagonists and strong, weak and partial agonists previously shown to elicit a wide range of biological effects [9]. The usefulness of studying the P14 TCR derives from the fact that T cells bearing this particular TCR have been extensively studied in murine models of autoimmunity, antiviral immunity and tumour rejection [4], [10–12]. Indeed, recognition of the index peptide by the P14 TCR can result in autoimmunity, antiviral immunity and tumour rejection while only some, if any, of these effects are mediated by the variant peptides. In addition, some of the peptide variants have been shown to induce positive and/or negative selection of P14 in fetal thymic organ cultures. The use of surface plasmon resonance (SPR) to measure off-rates and affinities for the P14 TCR and a range of related and well-characterised pMHC complexes offers a unique opportunity to examine the influence of off-rate and affinity on the biological consequences of T cell activation.

## Results and discussion

### T cell activation induced by related peptide ligands

Related peptide ligands derived from LCMV were tested for their ability to activate P14 transgenic T cells. T cell activation was measured by TCR down-regulation and by measuring the peptide concentration required for half-maximal proliferation (Table 1). Also, because of the likely influence of pMHC stability on T cell activation, we measured the stability of peptide binding by H-2D^b^ using an RMA-S stabilisation assay (Table 1). This group of peptides was chosen because their biological characteristics have been extensively documented previously [4, 10–14]. As shown in Table 1, the group includes strong agonists, weak and partial agonists and peptides that exhibit antagonist activity. Those peptides best at inducing TCR down-regulation are the strong agonists while antagonists are the least efficient at inducing TCR down-regulation. The next set of experiments was performed to determine whether binding of P14 to the different pMHC complexes correlated with T cell activation potency.

**Table 1:** P14 binding and T cell activation with various APL

Peptide	Sequence	TCR down-reg. (%) @ 10^–6^M	TCR-down-reg. (%) @ 10^–8^M	Half-max prolif. (M)	Peptide stability	Equil. *K*_D_	Δ*G*° (kcal/mol)	Agonist /antagonist	Ref(s)
A3V	KAAYNFATC	37	1	10^–7^	<1 h	1.4 µM	–8.0	Agonist	[9, 14]
M9C	KAVYNFATM	74	72	10^–11^	>1 h	2.3 µM	–7.7	Agonist	[18]
gp33-wt	KAVYNFATC	63	19	10^–8.5^	>1 h	3.5 µM	–7.4	Agonist	[9]
L6F	KAVYNLATC	22	11	10^–7.5^	>1 h	19µM	–6.4	Partial Agonist	[9, 11]
A4Y	KAVANFATC	15	4	10^–7.2^	<1h	18µM	–6.5	Partial Agonist	[4, 9]
mDBM	KAIYRFNAI	11	2	10^–5.8^	<1h	20µM	–6.4	Partial Agonist	[13]
mRPP	KALYDYAPI	0	1	10^–5^	∼1h	43µM	–6.0	Partial Ag./Antag.	[13]
C4Y	KAVCNFATC	–1	–1	>10^–5^	>1h	69µM	–5.7	Weak Ag./Antag.	[11]
S4Y	KAVSNFATC	3	3	0	>1h	70µM	–5.7	Antagonist	[13]
rDBM	KALYNYAPI	7	4	10^–7^	∼1h	73µM	–5.6	Partial Ag./Antag.	[13]
mNGF	VAVYRFNKY	0	0	10^–5^	>1h	92µM	–5.5	Partial Ag./Antag.	[13]
G4Y	KAVGNFATC	–2	–1	0	>1h	116µM	–5.4	Antagonist	[4, 9]
V4Y	KAVVNFATC	–1	0	>10^–5^	>1h	135µM	–5.3	Weak Ag./Antag.	[4]
AV	SGPSNTPPEI	0	1	0	>1h	NB	NB	Null	Null
rSCP	KAIFRFSAT	3	4	0	<1h	33µM	–6.1	Partial Agonist	[13]
rFAS	KAKYHGNVI	4	0	>10^–5^	>1h	11.5µM	–6.7	Partial Agonist	[13]

AV: adenovirus (negative control); gp33-wt: LCMV wild-type peptide antigen; mDBM: murine dopamine β-mono-oxygenase; mNGF: murine nerve growth factor; mRPP: murine retrovirus-related pol polyprotein; NB: no binding; rDBM: rat dopamine β-mono-oxygenase; rFAS: rat fatty acid synthase; rSCP: rat sodium channel protein.

### Binding of P14 TCR to H-2D^b^-gp33

In order to examine the binding of TCR to pMHC by Biacore SPR, it is necessary to construct artificially modified TCR that are stable in the soluble state. In order to achieve this, we expressed the extracellular domains and introduced a non-native disulphide bond. This strategy has previously been used to produce several heterodimeric TCR and does not result in any significant difference in affinity [15] or structure [16] compared with the same TCR produced by alternative, more established constructs. The kinetics of binding are also not affected by this non-native disulphide bond (D. Cole, unpublished data).

**Figure 1:**
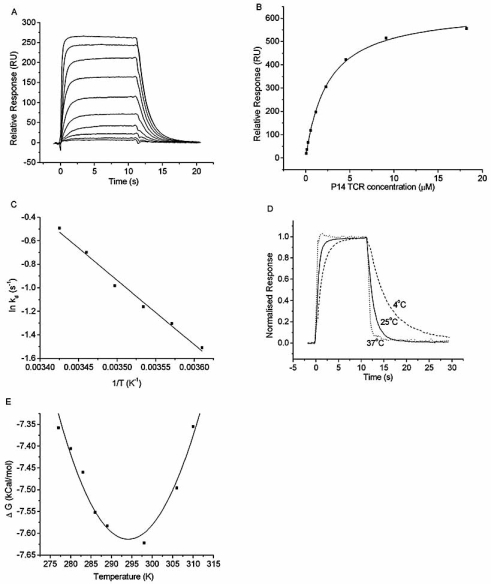
(A)Binding of P14 soluble TCR to H-2D^b^-gp33 peptide measured by SPR. Background responses to the control flow cell were subtracted and all data were plotted with the injection point at 0s. (B)Equilibrium binding of soluble P14 TCR to H-2D^b^-gp33 peptide. (C)Arrhenius analysis of dissociation kinetics for the P14 TCR binding to H-2D^b^-gp33; Arrhenius dissociation energy. ▵E_a_^diss^ = 13kcal/mol. (D)SPR binding of soluble P14 TCR binding to H-2D^b^-gp33 at various temperatures. (E)Plot of ▵*G* against temperature along with a least-squares fit of the equation ▵*G*°_T_ =▵*H*° +▵*C*_P_(*T*–*T*_0_) –*T*▵*S*° –*T*▵*C*_P_ln(*T*/*T*_0_) to provide an estimate of ▵*Cp*.

We first analysed binding of the P14 TCR to the index peptide, gp33, using a Biacore 3000 SPR instrument at 25°C (Fig. 1). We observed small amounts of irreversible non-specific binding even when conducting binding experiments immediately after gel filtration chromatography of the P14 TCR. This binding is likely due to very small quantities of denatured TCR aggregates. We observed preferential binding to the first flow cell regardless of the orientation of the test and control flow cells (data not shown), confirming the non-specific nature of aggregate binding. This artefact also becomes more apparent at high analyte concentrations (>5×*K*_D_), and at higher temperatures (probably due to the increased strength of hydrophobic interactions), making these data more difficult to analyse. We performed sequential injections with high TCR concentrations and did not observe any reduction in the binding response, indicating that this non-specific irreversible binding component does not interfere with specific TCR binding in these experiments

The P14 TCR binds to its cognate ligand with extremely rapid kinetics, but with an equilibrium affinity within the range previously observed for other antiviral TCR (3±0.5µM; Fig. 1B, Table 2). In particular, the off-rate (measured at 25°C) of approximately 0.975s^–1^ corresponds to a half-life (*t*_1/2_) of 0.71s – approximately 2–35 times faster than previously observed for other agonist ligands (reviewed in [17)]. Due to the very fast kinetics of binding, it was not possible to reliably measure P14 TCR on-rates, even at lower temperatures. We also measured P14 TCR binding at various temperatures, summarised in Table 2, and plotted the calculated Δ*G*° values against temperature to obtain values of Δ*H*° (the change in enthalpy) =–10.8kcal/mol, Δ*S*° (the change in entropy) =–8cal/mol and Δ*Cp* (the change in heat capacity) =–0.6kcal/mol. Since Δ*Cp*_conf_ cannot exceed the value of Δ*Cp* (because this would require the binding surface area to be negative), the maximum possible value of Δ*Cp*_conf_ is –0.6kcal/mol. This Δ*Cp* value is similar to previously reported TCR/pMHC interactions [8.].

**Table 2:** Binding parameters for P14 soluble TCR to H-2D^b^-gp33

Temperature (°C)	Equilibrium *K*_D_ (µM)	Off-rate, *k*_off_ (s^–1^)	Half-life (s)
4	1.55	0.221	3.1
25	2.42	0.975	0.71
37	6.48	n.d.	n.d.

n.d. = not determined

### 2.3 P14 TCR binding to altered peptide ligands

**Figure 2:**
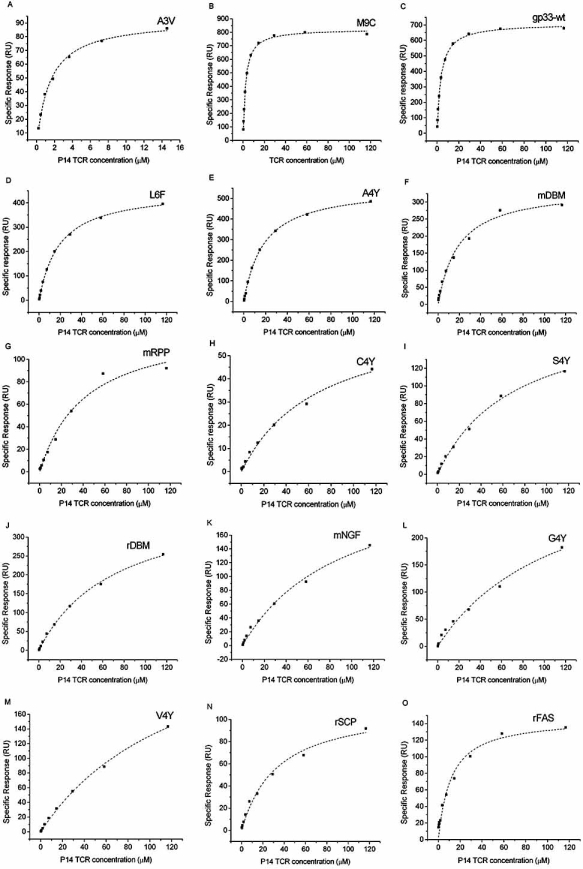
Equilibrium binding of P14 soluble TCR to H-2D^b^ complexed with various altered peptide ligands measured by Biacore 3000 SPR at 25°C. Peptide abbreviations as in Table 1.

**Figure 3:**
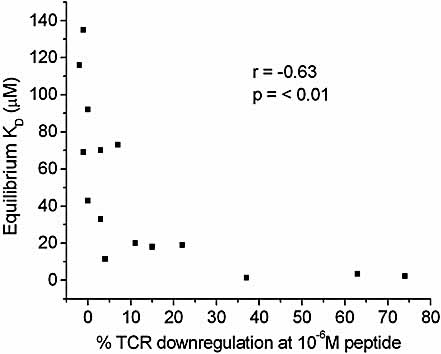
Binding affinities of P14 and the various APL were plotted against %TCR down-regulation observed upon stimulation with each peptide at 10^–6^M concentration. The correlation coefficient was calculated as 0.63, using Excel software (*p*<0.01).

In order to ensure that there was direct comparability between the measurements for various APL, we measured all of the APL binding data with a single preparation of P14 TCR. Fig. 2 shows the equilibrium binding curves for P14 TCR binding to various altered peptide ligands summarised in Table 1. Owing to the extremely fast kinetics, it was not possible to measure kinetic parameters for most of the APL, but in the few instances where off-rates could be estimated, they correlated with equilibrium dissociation constant measurements. It is likely that, with the extremely rapid on-rate we observed, this parameter is diffusion limited and changes in off-rate are therefore directly reflected by changes in *K*_D_ values. Many of the APL bind H-2D^b^ with very similar affinity to the wild-type peptide, and for these peptides, the binding affinities correlate with their T cell activation potency. For all other peptides, the relationship between their T cell activation potency and TCR binding affinity is accounted for by the stability of the pMHC complex; *e.g.* A3V is a relatively weak agonist due to the poor stability of the pMHC complex (<1h), whereas M9C is a potent agonist due to its much higher affinity for H-2D^b^ [18] causing a greater stability of its pMHC complex (>1h). Most unstable complexes (*t*_1/2_<1h) contained amino acid substitutions at position5, previously identified as an anchor residue for D^b^, although clearly there are exceptions since the lower affinity and reduced stability exhibited by peptide A3V are accounted for by the change at position3. Overall, however, binding affinity of the TCR/pMHC complexes was found to correlate well with T cell activation potency (*r*=0.63; Fig. 3). Indeed, TCR binding affinity and pMHC stability adequately account for the observed T cell activation potencies in this system.

### Thermodynamic measurements

We also measured changes in enthalpy (Δ*H*°), entropy (Δ*S*°) over the temperature range of 4–37°C and thus calculated the change in total heat capacity (Δ*Cp*). We have measured the affinity of the P14 TCR to various altered peptide ligands and find that the affinity of the TCR for each pMHC correlates well with the biological response to each of these peptides (Fig. 3). This study focused on the P14 TCR since previous *in vivo* studies have amply demonstrated the agonistic properties of this interaction in that recognition of H-2D^b^/gp33 by P14 transgenic T cells results in a wide range of biological effects such as autoimmunity [10, 11], tumour rejection [4] and antiviral immunity [19].

Our measurement of Δ*H*° of –10.8kcal/mol is similar to measurements made in other systems (*e.g.* –13kcal/mol for 2B4 binding to MCC [8]), whereas our Δ*S*° measurement of –8cal/mol is significantly smaller than the measurements of –20cal/mol for 2B4 TCR binding to MCC [8] and –51cal/mol for the JM22 TCR binding to HLA-A2-flu [20]. This implies that the amount of bond formation is similar, but the degree of ordering required is significantly lower for the P14 TCR binding to H-2D^b^-gp33. The relatively low Δ*S*° for P14 binding could therefore be responsible for the fast kinetics in this system. This is likely to be caused by relatively small amounts of molecular rearrangement upon P14 TCR binding, *i.e.* neither the TCR nor the pMHC need to significantly change their structures to enable successful binding, and water is also not significantly restructured, unlike in the LC13 TCR–B8-EBV system where Δ*S*° =+11.5cal/mol at least partially due to the release of structured water during binding [21].

To account for anomalies in the correlation between off-rates and T cell activation potency, ▵*Cp*_conf_ has been proposed as a parameter that contributes to determining T cell activation potency [22]. However, we found no evidence for this in the case of the P14 TCR, which displays a typical ▵*Cp* of –0.6kcal/mol (Fig. 1E), and ▵*Cp*_conf_ cannot exceed ▵*Cp* since this would require a negative interaction surface. A typical ▵*Cp* value excluding ▵*Cp*_conf_, calculated from various TCR/pMHC structures, is –0.25kcal/mol [21], implying that ▵*Cp*_conf_ for the P14 TCR binding is likely to be approximately –0.35kcal/mol.

### Discussion of activation models

Based on systems described in the literature, our finding that the P14 TCR/MHC-gp33 interaction has a half-life of only 0.7s is unexpected. The brevity of this interaction places it well below the spectrum of off-rates that have previously been observed for agonist ligands (comprehensively reviewed in [23]). Indeed, TCR/pMHC mean dwell-times of <1s have previously only been described for antagonist ligands. Thus, the P14/MHC-gp33 interaction extends the range of interactions that can result in T cell activation.

The kinetic proofreading model of T cell activation postulates that the off-rates of TCR/pMHC interactions correlate with their activation potencies. It is likely that the differences in affinity we observe between APL in the P14 system (Table 1) are the result of differences in the off-rate of the interaction. Thus, the order of potency within the ligands for the P14 TCR conforms to that predicted by kinetic proofreading. The difficulty comes when the P14 system is compared to other systems where ligands with half-lives of <1s act as antagonists or have no biological activity. While our data do not directly challenge the concept of kinetic proofreading, they do show that it is impossible to define an absolute TCR/pMHC off-rate at 25°C for T cell activation across all systems. Thus, our findings suggest that, at a minimum, the concept of kinetic proofreading requires further refinement to account for the >10-fold difference between agonist TCR/pMHC off-rates in different systems. We are unable to determine what other factors might account for these differences from our study of the interaction of soluble molecules in three dimensions by SPR. However, it is very likely that the outcome of antigen engagement at the cell surface will depend on the particular context of the TCR that is being engaged.

There are several ways that this context could vary between T cells with different TCR, which might explain why the TCR/pMHC off-rates measured by SPR for a particular TCR from one T cell might not bear any direct relationship to the off-rates measured by SPR for another system. Firstly, the temperature at which Biacore SPR is performed is 25°C (the ‘standard’ physical temperature), whereas the temperature of T cell activation assays is 37°C. However, it seems unlikely that TCR/pMHC interactions would disproportionately vary between these temperatures between different systems. Indeed, all TCR/pMHC interactions seem to increase in their rapidity at 37°C compared to 25°C (Fig. 1D), and by similar extents (D. Cole, unpublished data). Secondly, Schamel *et al.* [24] have recently discovered that the TCR on a given T cell can vary in their multivalency. This could occur systematically between different T cells with different TCR, leading to varying levels of sensitivity between the different T cells. The avidity effect of multivalent TCR may enable some T cells to compensate and activate in response to weaker or shorter interactions than others. Thirdly, the level of preformed TCR/coreceptor complexes on the T cell surface, thought to be better at responding to antigen than uncomplexed TCR, may vary [25]. Thus, both the level of multivalent aggregates and the level of TCR/coreceptor complexes can differ between T cells, and could explain why the off-rates of two ligands that are potent activators of different T cells measured by SPR might bear no obvious relationship. Fourthly, it is possible that TCR/pMHC interactions may be differentially affected by the mechanical stress induced by T cell-APC interaction [23]. Finally, it is likely that the pMHC concentrations on the surface of APC vary between different T cell specificities. We have measured the stability of the various peptides in the H-2D^b^ MHC molecule (Table 1), but it is also likely that variations in peptide processing efficiency may influence this parameter to a significant degree. Very few peptide antigens have been accurately measured for their level of cell surface expression, but the range of reported peptide antigen levels is from ∼300copies/cell down to ∼8copies/cell [26]. It is possible that T cells responding to more abundant peptides may tolerate faster kinetics than those responding to scarcer peptides.

## Concluding remarks

In conclusion, we report that binding of the P14 T cell receptor to its cognate ligand occurs with the fastest kinetics reported for an agonist TCR to date, yet all other thermodynamic parameters are within the range previously observed for TCR/pMHC interactions. This observation significantly broadens the range of off-rates that have been observed for TCR binding to immunodominant agonist peptides. Although the data for P14 TCR binding to altered peptide ligands do not contradict the kinetic proofreading model of T cell activation, they certainly show that it is difficult to define a single parameter that governs T cell activation for all T cells. More questions will be answered by studying engineered TCR with defined properties produced by the recently reported TCR phage display technology [27], *e.g.* TCR with faster or slower kinetics but with similar equilibrium *K*_D_.

## Materials and methods

### TCR down-regulation

Spleen cells from transgenic mice were mixed with peptide-pulsed macrophages, centrifuged and incubated at 37°C in IMDM supplemented with 10% FCS in round-bottom 96-well plates. Macrophages were pulsed with the different peptides (10^–6^M) for 1h at 37°C and subsequently washed twice. After 4h, the cells were harvested and stained with CD8- and Vα2-specific antibodies before analysis by flow cytometry.

### Binding assays

MHC classI stability assays were performed using the TAP-deficient cell line RMA-S [28]. RMA-S cells were washed once in PBS and cultured in serum-free RPMI containing 100µM peptide. After a 2-h incubation at 37°C, the cells were washed three times in cold RPMI. The cells were resuspended in 1mL serum-free RPMI and incubated for 0, 30, 60 and 90min at 37°C. Aliquots of 2×10^5^cells were removed at each time point for staining with a FITC-conjugated conformation-dependent H-2D^b^-specific antibody (Pharmingen). Subsequently, the cells were resuspended and fixed in PBS containing 0.5% paraformaldehyde and analysed by flow cytometry using CellQuest software. The half-life of the MHC-peptide complexes was measured by calculating the fluorescence index (FI) at each time-point. FI =(mean fluorescence sample – mean fluorescence background)/(mean fluorescence no peptide – mean fluorescence background).

## Expression of P14 soluble TCR

TCRα and β cDNA was a generous gift from Prof. Hans-Peter Pircher (Freiburg, Germany). Overlapping PCR was used to introduce the following mutations (IMGT numbering/nomenclature: http://imgt.cines.fr): TRAC01 threonine49 → cysteine, TRBC01 serine56 → cysteine, TRBC01 cysteine70 → serine [15]. This introduces an inter-chain disulphide bond in the folded protein while removing the free cysteine in the βchain. TCR α and βchains were truncated immediately prior to the membrane-proximal cysteines and were cloned into the pGMT7 expression vector to produce pJMB098 and pJMB099, respectively [15].

Modified TCR α and βchains were expressed separately as insoluble inclusion bodies in *E.coli* strain BL21-DE3 (Novagen). Inclusion bodies were washed several times, then denatured in 6M guanidine-HCl, 10mM DTT, 2mM EDTA, 50mM Tris pH8.1, and quantified using a Coomassie binding assay (PerBio). Using 30mg of each TCR chain, refolding was performed by rapid dilution into 1L of 5M urea, 0.4M l-arginine-HCl, 0.1M Tris pH8.1, 2mM EDTA, 6.5mM cysteamine, 3.5mM cystamine. Following overnight incubation at 4°C, the mixture was dialysed twice against ten volumes of 10mM Tris pH8.1. Refolded soluble TCR was then purified by anion exchange chromatography on a 5-mL QFF column (Amersham). Soluble P14 TCR eluted first at approximately 150mM NaCl and was concentrated to approximately 1mL by centrifugal concentration (Amicon centriprep) prior to size-exclusion chromatography on a Superdex 200 HR column (Amersham) equilibrated in 10mM Hepes pH7.4, 150mM NaCl, 3mM EDTA, 0.01% P-20. P14 soluble TCR eluted at approximately 15.5mL and was concentrated to approximately 5mg/mL by centrifugal concentration (Amicon centricon). TCR concentration was measured by spectrophotometry at 280nm wavelength using an extinction coefficient calculated from the protein's amino acid sequence (=75450M^–1^cm^–1^).

### Biacore SPR binding analysis

Biotin-tagged H-2D^b^-peptide complexes were made by *in vitro* refolding with synthetic peptide as described previously [29], except that *in vitro* biotinylation was not performed and complexes were immobilised by the natural level of specific biotinylation which occurs during expression of proteins containing a BirA recognition sequence at their Ctermini [30]. A Biacore 3000 machine was run at 25°C and 5µL/min flow rate. At least 2000RU of streptavidin was immobilised, by amine coupling, to each flow cell on a CM-5 chip. Approximately 1000RU H-2D^b^-peptide complex was immobilised on each flow cell. Soluble P14 TCR was diluted to the concentrations indicated and injected over the chip surface using the appropriate injection program. TCR dissociated rapidly from the H-2D^b^-peptide-coated chip surface at the end of each injection, so that no regeneration phase was required. Binding response measurements were corrected for non-specific control chip responses used to determine equilibrium and kinetic binding parameters, using Biacore Biaevaluation software. Equilibrium binding constants (*K*_D_) were determined using the Origin6.0 program (Microcal) by least-squares fitting to the 1:1 Langmuir binding equation: *R*=*R*_max_ ×[TCR]/(*K*_D_ +[TCR]), and confirmed by linear Scatchard analysis.

Kinetic analyses were performed at a flow rate of 50µL/min and at a data collection rate of 5Hz. Data were analysed using the Biacore Biaevaluation software and were plotted using the Origin6.0 program (Microcal). Δ*G* values were calculated from *K*_D_ values using the equation Δ*G*° =–ln*RT*.*K*_D_ and were plotted against temperature using the Microcal Origin6.0 program. Least-squares fitting was performed using the equation ▵*G*°_T_ =▵*H*° +▵*C*_P_(*T*–*T*_0_) –*T*▵*S*° –*T*▵*C*_P_ln(*T*/*T*_0_) [8].

## Abbreviations

APL: altered peptide ligand
FI: fluorescence index
LCMV: lymphocytic choriomeningitis virus
pMHC: peptide-MHC complex
SPR: surface plasmon resonance
